# Multilayer Graphene Nanoshells from Biomass for Fast-Charge, Long-Cycle-Life and Low-Temperature Li-Ion Anodes

**DOI:** 10.3390/ma18163918

**Published:** 2025-08-21

**Authors:** Kevin R. McKenzie, Nathan A. Banek, Michael J. Wagner

**Affiliations:** Department of Chemistry, The George Washington University, Washington, DC 20052, USA

**Keywords:** biochar, valorization, carbon onions, laser pyrolysis

## Abstract

Graphene nanoshells (MGNS) were prepared from cellulose, a sustainable biopolymer. Different sizes/morphologies were obtained by simply changing the metal catalyst salt in the synthesis. The MGNS were shown to reversibly cycle Li-ions by an intercalation mechanism similar to graphite. The reversible capacity of each MGNS prepared from different metal salts correlates well to its degree of 3-D graphitic order. The small size of the MGNS allows for short Li diffusion distances and very rapid charging, obtaining a 20% charge in 36 s (100 C rate). The unique spherical structure provides stable cycling, losing only 3.8% capacity over 900 cycles, and eliminates exfoliation that occurs when cycling graphite in propylene carbonate (PC), an inexpensive, environmentally friendly electrolyte. This enables cycling in a PC-only solvent-based electrolyte, with stable cycling and high capacities at temperatures as low as −35 °C. At this very low temperature, 95% of the RT reversible capacity is retained, with only a modest charge potential increase due to the increase in viscosity of the solvent.

## 1. Introduction

Today’s market for batteries to power electric vehicles (EVs) is dominated by lithium ion (Li-ion) technologies [1]. Li-ion batteries employ a “rocking chair” mechanism in which Li^+^ is shuttled between intercalation materials that comprise the anode and cathode. The use of intercalation materials as anode active material rather than lithium metal is necessitated by safety concerns but decreases the specific capacity significantly, 372 mAh/g for graphite which is used in the vast majority of commercial cells [2]. However, the production of Li-ion battery graphite is energy intensive and causes severe damage to the environment. While alternative green synthetic methods have been shown to produce graphite that can match the performance of commercial Li-ion battery graphite [3,4,5], they have not been implemented for large scale production to date. Furthermore, graphite has additional disadvantages including modest rate capability and the inability to reversibly cycle in inexpensive low temperature electrolyte solvents including propylene carbonate (PC) [6,7,8]. The inabilities of Li-ion cells to charge rapidly and perform at sub-zero temperature are severe limitations for EVs; vehicles powered by internal combustion engines have greater driving ranges, can be refueled in a fraction of the time and perform well in northern climates [9].

It has been shown that replacing graphite with carbon nanospheres (HCNs) can dramatically increase cell rate capability due to their high surface areas and small particle size. Although HCNs exhibit unacceptably low first cycle Coulombic efficiencies, typically 30–68%, excellent long-term full cell performance can be achieved by pre-litiation. However, HCNs reported to date have been synthesized from relatively high cost or non-renewable resources, generally by poorly scalable methods [10], and have a turbostratic or amorphous structure, yielding a sloping discharge curve rather than the potential plateau typical of graphite, resulting in lower discharge potential [9,11,12,13,14].

Here we show that a carbon negative material made from biomass, multilayer graphene nanoshells (MGNS) [15], exhibits electrochemical properties that are similar to graphite but can be charged (loaded with Li) much faster and cycles stably at temperatures as low as −35 °C with good capacity in propylene carbonate, an inexpensive, environmentally benign solvent that is incompatible with industry standard graphite anodes. In addition, MGNS anodes exhibit long cycle lives even when charged and discharged at very high rates.

## 2. Experimental Section

### 2.1. MGNS Synthesis

Details of MGNS synthesis have been provided in previous publications [16,17]; thus, only a brief description is included here. Salts of NiCl_2_·6H_2_O (ReagentPlus, Sigma Aldrich, St. Louis, MO, USA) and CoCl_2_·6H_2_O (99.9%, Alfa Aesar, Ward Hill, MA, USA) were dehydrated by heating at 150 °C for 2 h under dynamic vacuum, while FeCl_2_ (Anhydrous 99.5%, Alfa Aesar) was used as received. Typically, a 4:1 mass ratio of cellulose (Avicel PH-105 NF, FMC BioPolymer, Philadelphia, PA, USA) and metal chloride salt were mixed by ball milling, as previously described. The resulting powder was pressed (10.89 t, Carver 3851 benchtop laboratory press, Bloomfield, CT, USA) to form 20 mm diameter, ~4 g pellets. A hole was then drilled in the center of each pellet followed by pyrolysis (N_2_ gas, 30 mL/min) from room temperature to 375 °C at a 10 °C/min ramp rate with 30 min soak time. The charred pellets were irradiated by a 2 mm diameter 10.4 µm wavelength laser beam (Firestar t60, Synrad Inc., 95% power, Mukilteo, WA, USA) or a 200 W fiber coupled laser (1080 nm, BWT, Singapore) while rotating on a ¼ stainless steel rod at a linear velocity of 1.63 mm/s (1.2 rev/min) under vacuum (0.5 torr He) for one full rotation. The raw product was purified by microwave digestion, heating from room temperature to 210 °C in 10 min and held for an additional 30 min in HNO_3_ solution (ACS Grade, 68–70% HNO_3_) using a XP-1500+ Teflon vessel and MARS 5 Digestion Microwave System (CEM Corp., Matthews, NC, USA). After cooling to room temperature, the mixture was diluted with of deionized water and the solid product collected by vacuum filtration (1 µm polyster, GVS LifeSciences, Bologna, Italy). The product was then washed with additional deionized water until a neutral pH was obtained, rinsed with 1 M NaOH (>97% Fisher Scientific, Waltham, MA, USA) followed by deionized water neutralization, rinsed with a 10 *v*/*v*% HCl solution followed by deionized water neutralization and finally dried under vacuum. The mass yield after drying was 32%, 29%, and 40% of the C present in the char using Ni, Fe and Co chloride, respectively, as the catalyst.

### 2.2. Electrode and Electrochemical Cell Preparation

MGNS composite material (80 mg), carbon black (10 mg, Super C45, TIMCAL America Inc., Westlake, OH, USA), 5% Li-polyacrylate binder solution (200 µL) prepared by dissolving poly(acrylic acid) (1000 kDa, Polysciences, Warrington, PA, USA) in deionized water and neutralizing with LiOH (95%, Strem, Newburyport, MA, USA), and ethanol (20 µL, 200 proof, Pharmco-Aaper, Brookfield, CT, USA) were mixed with a Pulverisette 23 MiniMill (Fritsch Milling, Pittsboro, NC, USA) using a stainless steel cup (10 mL) and six balls (5 mm diameter) at 50 Hz for 15 min to form a slurry. The slurry was cast onto copper foil (0.127 mm, 99.9%, Alfa Aesar) and dried under vacuum at 150 °C for 2 h, resulting in areal loadings of ~2 mg/cm^2^. Round electrodes (16 mm diameter) were cut from the resulting sheet using a die cutting press (MSK-T-07 Precision Disc Cutter, MTI Inc., Salt Lake City, UT, USA).

Coin cells (CR2016, MTI Inc.) were assembled in an Ar filled dry box (<0.1 ppm O_2_ and H_2_O) with MGNS and Li metal (99.9%, MTI Inc.) electrodes separated by a polypropylene porous membrane (Celgard 3401, Charlotte, NC, USA). The electrolytes used were 1 M LiPF_6_ in EC:DMC (1:1 *v*/*v*) mixture (battery grade, <15 ppm H_2_O content, Sigma Aldrich) with 10% FEC (>99%, Solvay, Brussels, Belgium) by volume and 1 M LiPF_6_ in propylene carbonate (PC, battery grade, <15 ppm H_2_O content, Sigma Aldrich, Burlington, MA, USA).

### 2.3. Electrochemical Analysis

All electrochemical cycling was performed with an Arbin Instruments BT2000 (College Station, TX, USA) except for high-rate cycling, which was performed with a Maccor Inc. MC-4 (Tulsa, OK, USA). Unless otherwise stated, cells were cycled between open circuit voltage and 0.02 V then under reverse current to 1.5 V, at a C/4 rate. Cells were rested for 15 min between load and unload.

### 2.4. Characterization

Powder XRD patterns were obtained with a Rigaku Miniflex+ (Tokyo, Japan) with a zero-background oriented quartz crystal sample holder. Surface area and porosity measurements were obtained with a Micromeritics Instrument Company Tri-Star 3000 (Norcross, GA, USA). Samples were degassed at 150 °C under vacuum and the saturation vapor pressure of N2 was measured concurrently with each sample measurement. An FEI Talos 200 kV with a field-emission source was used to obtain TEM and elemental composition (EDX, Lanham, MD, USA). Raman spectra were acquired with a LabRAM HR Evolution microscope (Horiba, Japan) with a 532 nm diode laser. Therogravimetric (TGA) analysis was performed on a Perkin-Elmer PYRIS 1 (Shelton, CT, USA), heating in air at a rate of 20 °C/min.

## 3. Results and Discussion

### 3.1. Biochar

During the charring process, bio-oils and gas evolved from cellulose leaving biochar as a hard black material. Numerous peaks were detected by XRD in each sample, all of which can be attributed to the metal salts, consistent with the cellulose decomposing into amorphous polycyclic aromatic hydrocarbons as expected from previous literature reports [18,19].

### 3.2. Unpurified Product Made with 20% w/w Metal Salt

**Figure 1:**
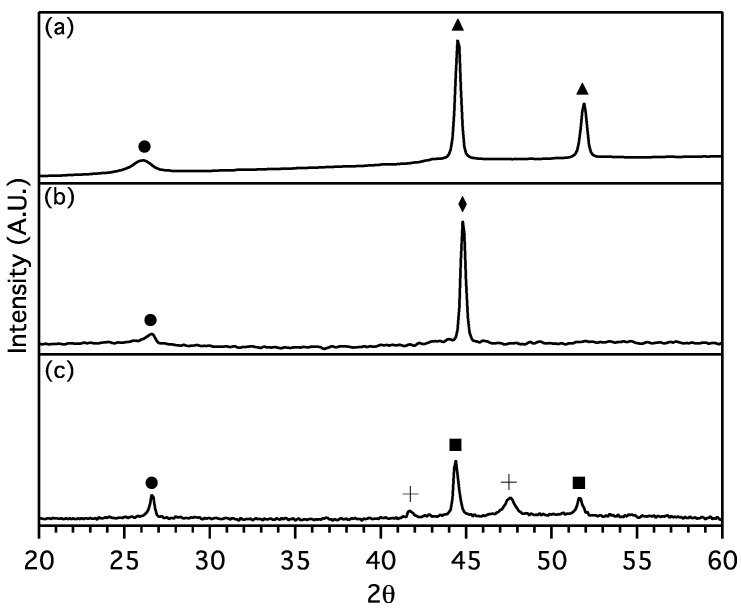
XRD patterns of post-irradiated raw products prepared from (**a**) NiCl_2_ (Ni labeled with triangles, JCPDS #04-0850), (**b**) FeCl_2_ (Fe labeled with diamond, JCPDS #06-0696), and (**c**) CoCl_2_ (FCC Co labeled with squares, JCPDS #15-0806, and HCP Co labeled with crosses, JCPDS #01-1278). Graphite 2H is labeled with circles, JCPDS #41-1487.

Powder X-ray diffraction patterns show no evidence of metal salts after laser irradiation, instead replaced by the patterns of metallic Ni, Fe, and Co (Figure 1). Cubic and hexagonal Co is evident, while Ni and Fe are present in single, cubic phases. In addition, a peak at 26° 2-theta appears in each sample very close to the d-spacing expected for the graphite 2H (002) reflection. Apparently, laser pyrolysis reduced the metal chloride salt to metal while catalyzing the conversion of amorphous carbon into a material with considerable graphitic character.

**Figure 2:**
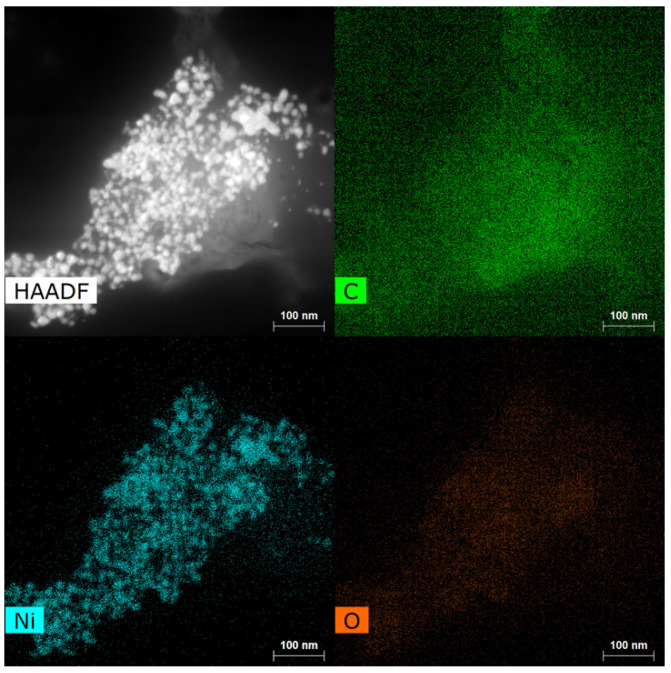
Raw product from NiCl_2_ HAADF image and EDS HyperMap of (C), (Ni), and (O).

**Figure 3:**
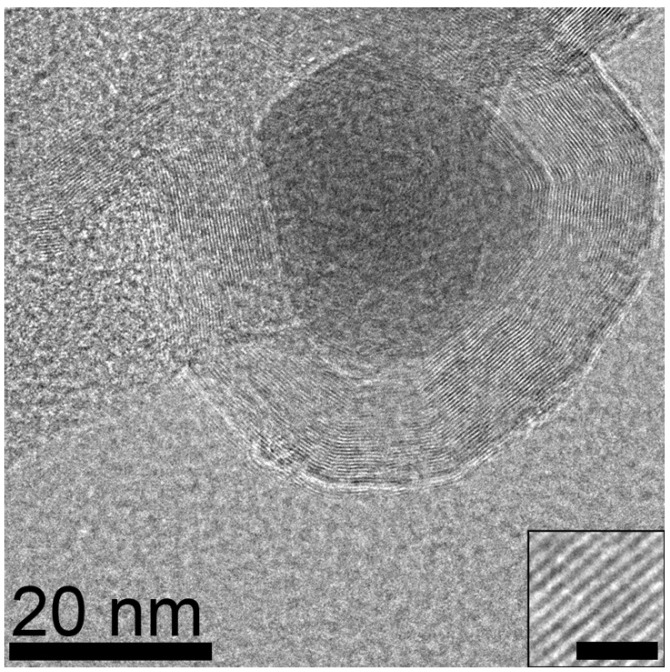
TEM micrograph of Ni particle in raw product encapsulated by multilayer graphene shell. Inset shows graphene layer spacing (scale bar is 2 nm).

Elemental maps (EDS) recorded for samples synthesized from NiCl_2_ revealed Ni nanoparticles ranging from 10 to 30 nm embedded in a carbon matrix (Figure 2). At higher magnification the Ni nanoparticles can be seen as fully encapsulated in a ~10 nm shell of graphene layers. The spacing between the graphene layers was found to be 3.4 Å, consistent within the precision of the measurement with the *d*_002_ spacing expected for graphite (Figure 3). The low oxygen signal in the EDS indicates that the nickel is well protected by the graphene shell from atmospheric oxidation.

**Figure 4:**
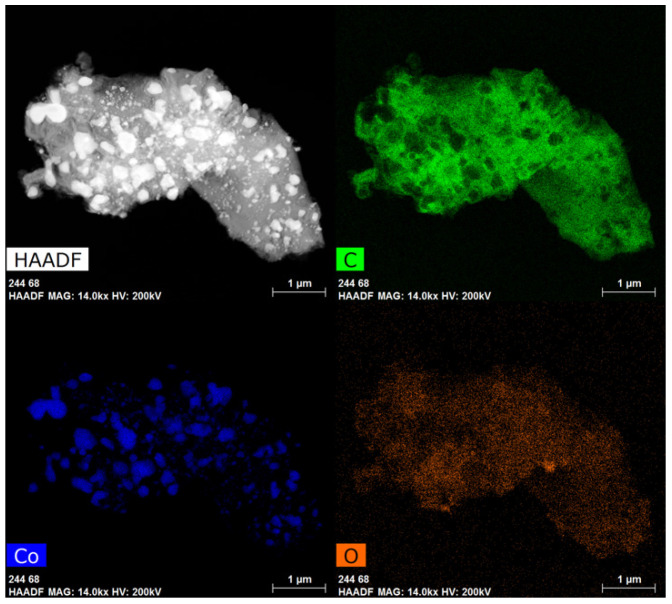
Raw product from CoCl_2_ HAADF image and EDS HyperMap of (C), (Co), and (O).

**Figure 5:**
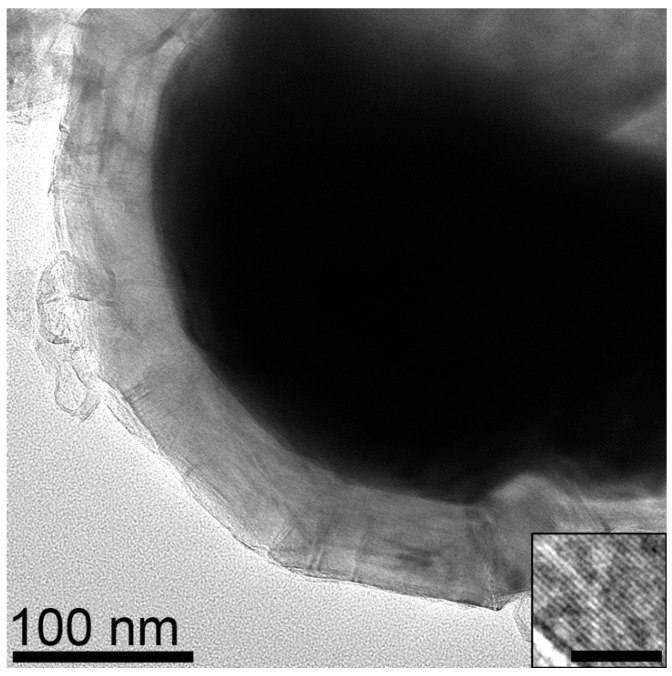
TEM micrograph of Co particle in raw product encapsulated by multilayer graphene shell. Inset shows graphene layer spacing (scale bar is 5 nm).

EDS maps of samples from CoCl_2_ consist of Co particles ranging from 130 to 400 nm with an average size of 250 +/− 80 nm as well as some smaller particles ranging from 30 to 80 nm in diameter (Figure 4). Closer analysis of encapsulated Co particles reveals that the graphene layer shell thickness is ~55 nm (Figure 5). The Co is also fully encapsulated in the graphene shell as evidenced by the lack of correspondence of the location of Co with O on EDS maps.

**Figure 6:**
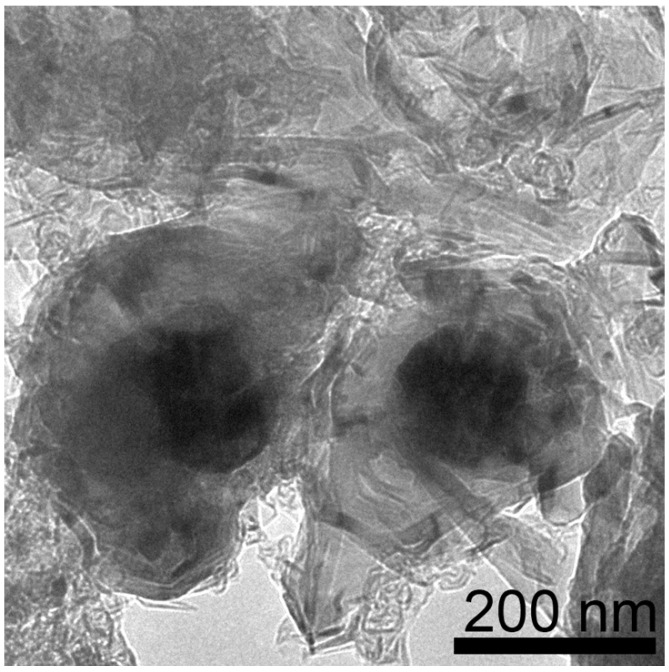
TEM micrograph of Fe particles in raw product encapsulated by multilayer graphene shells.

**Figure 7:**
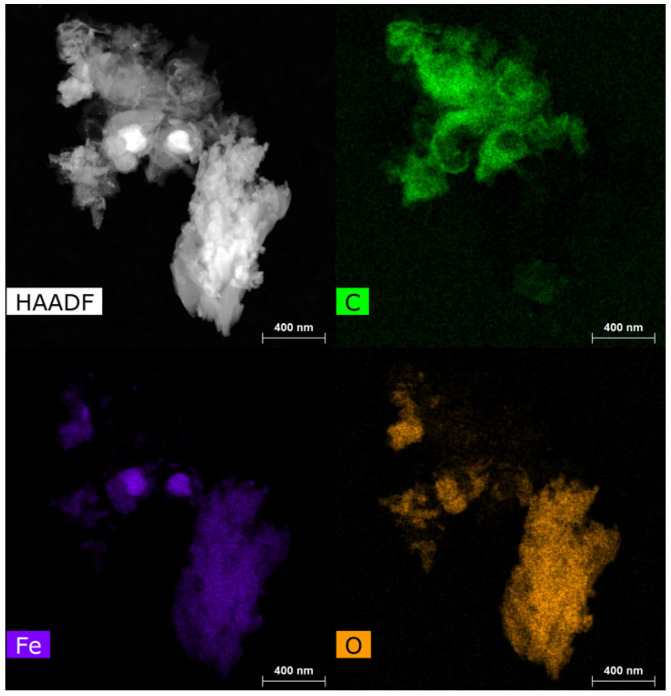
Raw product from FeCl_2_ HAADF image and EDS HyperMap of (C), (Fe), and (O).

EDS maps of samples produced from FeCl_2_ show Fe particles ranging from 100–250 nm and what appears to be numerous agglomerated multilayer graphene sheets 5 to 20 nm thick (Figure 6). Some Fe particles appear to be wrapped in graphene, but the large amount of surface oxide present on the metal evident in EDS maps indicate that they are only partially encapsulated and many heavily oxidized, uncoated iron particles are present as well (Figure 7).

### 3.3. Purified Product Made with 20% w/w Metal Salt

**Figure 8:**
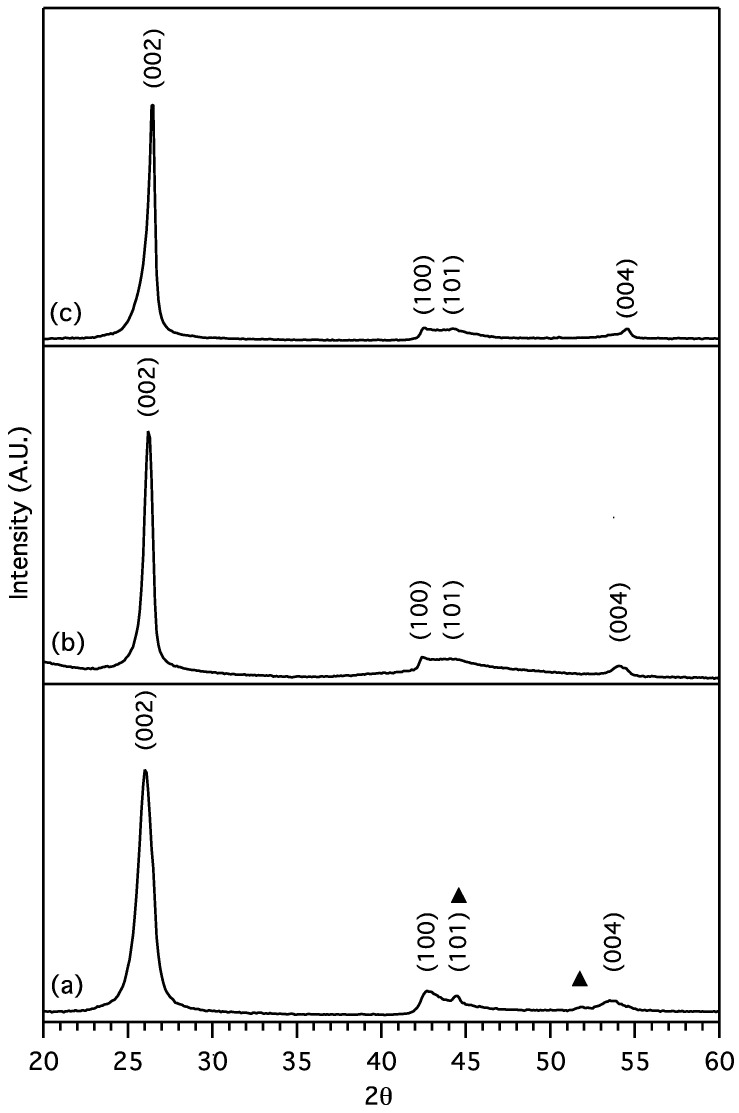
XRD patterns of MGNS from (**a**) NiCl_2_ with Ni impurities (triangle), (**b**) FeCl_2_, (**c**) CoCl_2_.

Powder X-ray diffraction patterns of purified MGNS from each metal salt in Figure 8 have broad peaks at ~26, 43, 44.5 and 54° with similar positions and relative intensities to graphitic (002), (100), (101), and (004) planes, respectively. Crystallite sizes were determined by Scherrer’s equation [20] using shape factors (K) of 0.9 and 1.84 for the c-axis (Lc) and a-axis (La) crystallite sizes, from the (002) and (100) peak full-width-at-half-maximums (FWHM), respectively.

**Figure 9:**
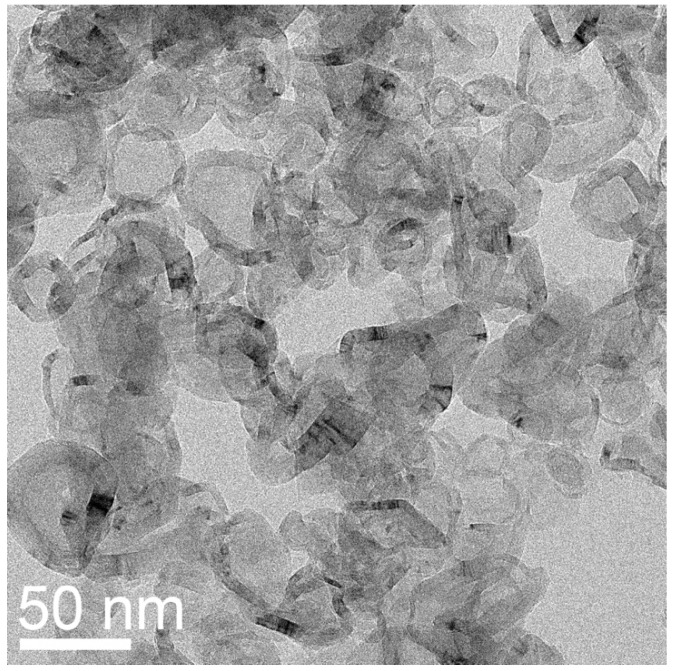
TEM micrograph of a typical agglomerate of MGNS-Ni showing spheroidal structure.

**Figure 10:**
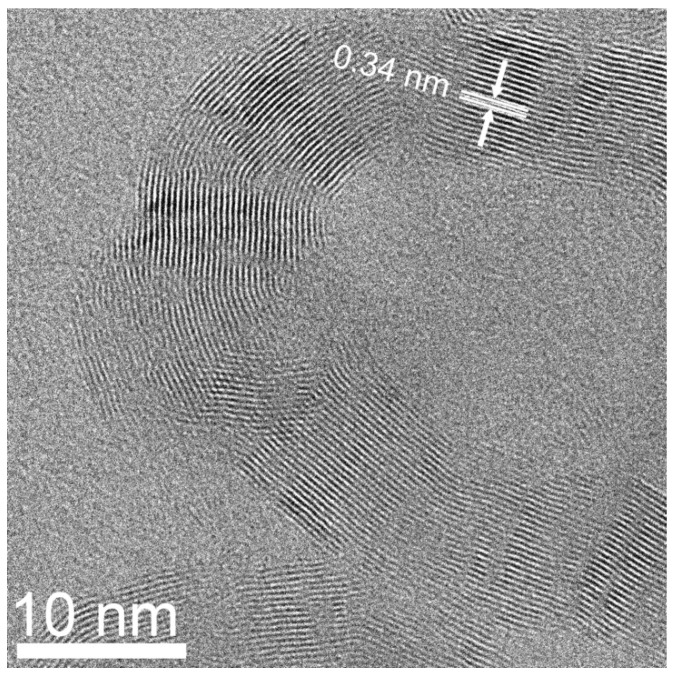
HR-TEM micrograph of MGNS-Ni showing the layered graphene structure. The superimposed arrows and dimension lines show the interwall spacing.

For the purified sample prepared from NiCl_2_, denoted as MGNS-Ni, crystallite sizes were found to be 9.4 nm (Lc) and 23.4 nm (La). The (002) peak position is centered at 26.0° 2-theta which corresponds to a d-spacing of 3.42 Å, significantly larger than expected for 3-D crystalline graphite, 3.354 Å (Figure 8a). TEM micrographs show agglomerations of 30 to 50 nm diameter spheroidal shells (Figure 9). HR-TEM images reveal the multilayer graphene structure of these shells with interlayer spacing of 3.4 Å, in good agreement with XRD results (Figure 10).

The large d-spacing indicates a high degree of turbostratic disorder or equivalently a low degree of 3D graphitic order, which can be estimated to be 23% using the formula [21].

(1) $$g=(3.44-d_{002})/(3.44-3.354)$$

where *g* is the degree of graphitization and *d*_002_ is the average interlayer spacing. Given the curvature of the shells, a high amount of turbostratic disorder is not surprising; graphene layers are staggered in crystalline graphite, allowing “nesting” of the layers and closer spacing, which is not possible for nanoshells because, assuming that the graphene carbon-carbon bond length remains the same, every shell must have a different number of atoms, necessitating some degree of lattice mismatch. TEM images (Figure 9) show that the MGNS are distorted spheroids with a significant fraction of the walls lacking curvature, accounting for some degree of 3D graphitic order and a relatively large La value.

The highly oxidizing environment during purification extracted most of the Ni leaving behind MGNS. Holes can be observed in the walls of many of the MGNS, presumably where the metal was extracted. A small number of encapsulated Ni particles were observed on the TEM grid and minor Ni impurities can also be observed in the XRD pattern. The residual Ni was determined to be 1% wt. by TGA (Figure S1), which equates to one encapsulated nickel per ~110 empty MGNS, in good agreement with EDS and TEM observations, calculated by using 50 nm as the MGNS diameter, 10 nm as the graphene wall thickness, and the bulk densities of Ni and graphite.

**Figure 11:**
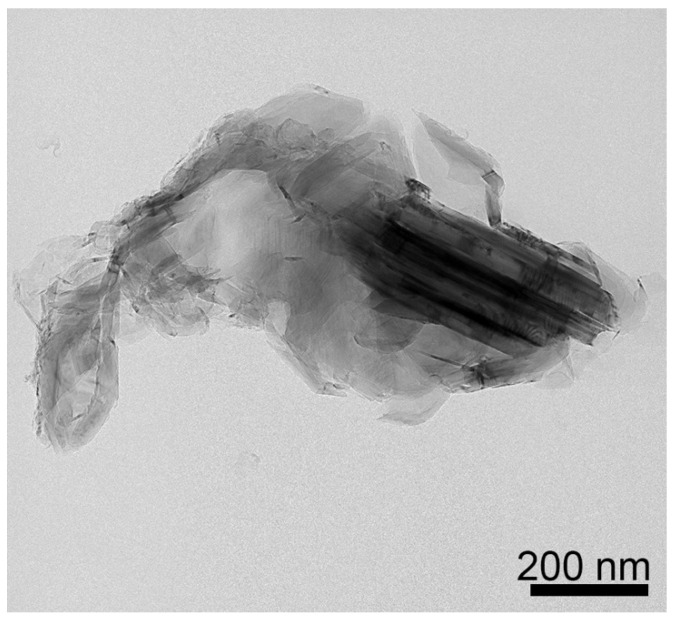
TEM micrograph of MGNS-Fe showing co-existence of multilayer graphene sheets and shells.

**Figure 12:**
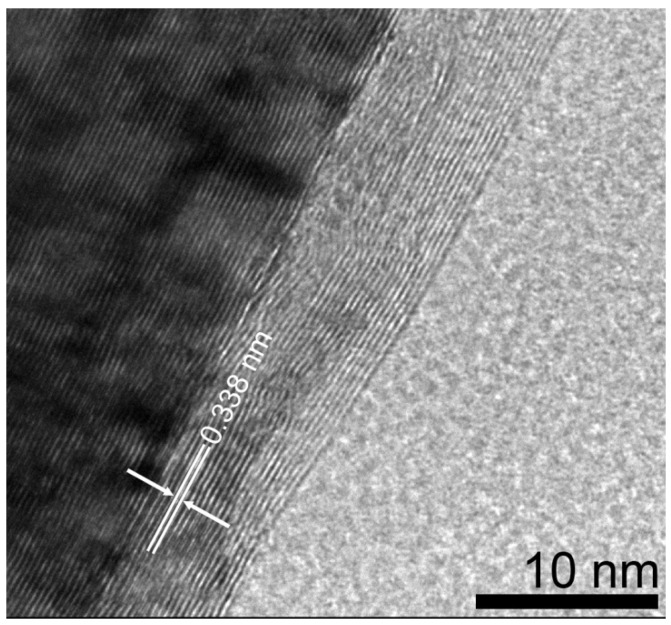
HR-TEM micrograph of MGNS-Fe showing the layered graphene structure. The superimposed arrows and dimension lines show the interwall spacing.

The (002) peak for MGNS made with FeCl_2_ appears at 26.2° 2-theta, yielding an interlayer spacing of 3.39 Å, with crystallite sizes of 16.1 nm (Lc) and 26.0 nm (La) (Figure 8b). Unlike MGNS-Ni, the majority of sample consisted of agglomerates of multilayer graphene sheets (Figure 11). Cavities in the agglomerates can be seen that are consistent with the Fe particle occupation sites observed prior to removal with acid. Graphitic lattice fringes are observable at high resolution, with interlayer spacings (3.38 Å) that are consistent with XRD data (Figure 12). It appears that Fe catalysts result in multilayer graphene growth rather than MGNS.

**Figure 13:**
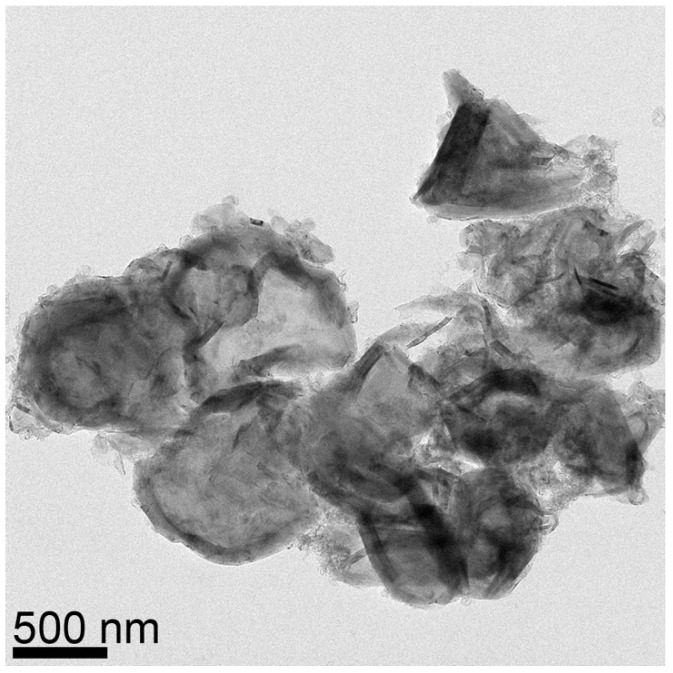
TEM micrograph of MGNS-Co agglomerate after purification.

**Figure 14:**
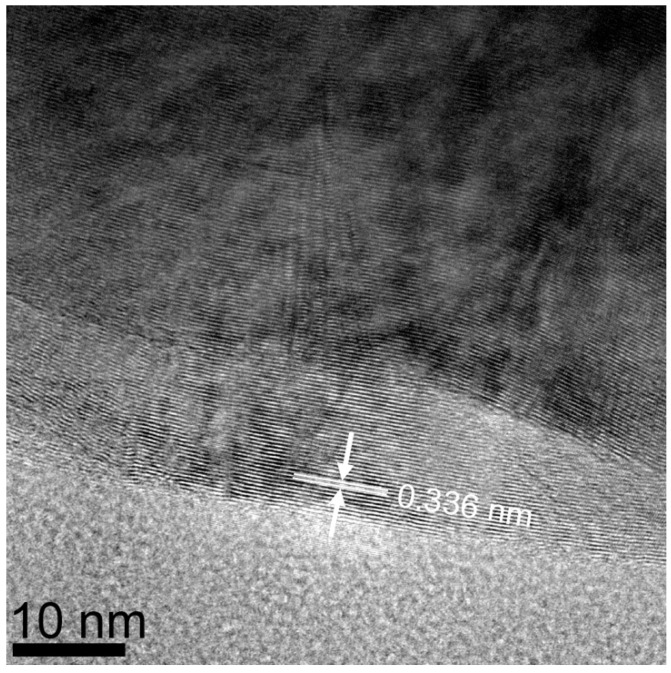
HR-TEM micrograph of MGNS-Co showing the layered graphene structure. The superimposed arrows and dimension lines show the interwall spacing.

Synthesis with CoCl_2_ (MGNS-Co) results in large hollow shells, 300 to 1 µm in diameter (Figure 13). The shells, with the metal centers removed, appear to be flexible, able to distort to a greater degree than MGNS-Ni. Many of the shell’s walls are sharply bent in places, with spans of the walls having very little curvature. The layers are highly ordered, resembling a graphitic structure when observed at high magnification (Figure 14). The larger shell size allows the graphene shells to more closely nest; the interlayer spacing, determined from the (002) XRD peak position (26.5° 2-theta), is 3.36 Å, indicating low turbostratic disorder (93% 3D graphitic order), much lower than found for either MGNS-Ni or MGNS-Fe (Figure 8). The crystallite sizes were determined to be 32 nm (Lc) and 31 nm (La) from XRD data, much larger than those of MGNS-Ni or MGNS-Fe. The thermal stability in air is very similar to Ni-MGNS (Figure S1).

### 3.4. Surface Area and Porosity

**Figure 15:**
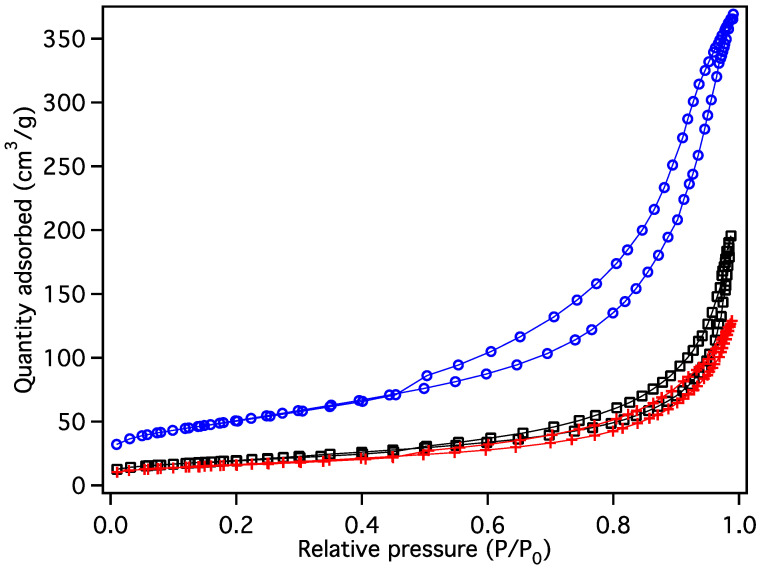
Nitrogen isotherm data for purified graphitic carbons from CoCl_2_ (red crosses), FeCl_2_ (black squares), NiCl_2_ (blue circles).

The BET surface area was measured by N_2_ adsorption and found to be 155, 70 and 60 m^2^/g for MGNS-Ni, MGNS-Fe, and MGNS-Co, respectively (Figure 15). The surface area decreases as the average shell size of each material increases, as one might expect. Hysteresis between N_2_ adsorption and desorption is notably present in the isotherm curve for MGNS-Ni indicating capillary condensation. A small degree of hysteresis is observed in MGNS-Fe indicating some shells are present, but the presence of multi-layer graphene also contributes to the adsorption/desorption behavior. The MGNS-Co has no notable hysteresis; its macroporous morphology (>100 nm) is too large for N_2_ adsorption/desorption to accurately quantify.

### 3.5. Raman Spectroscopy

**Figure 16:**
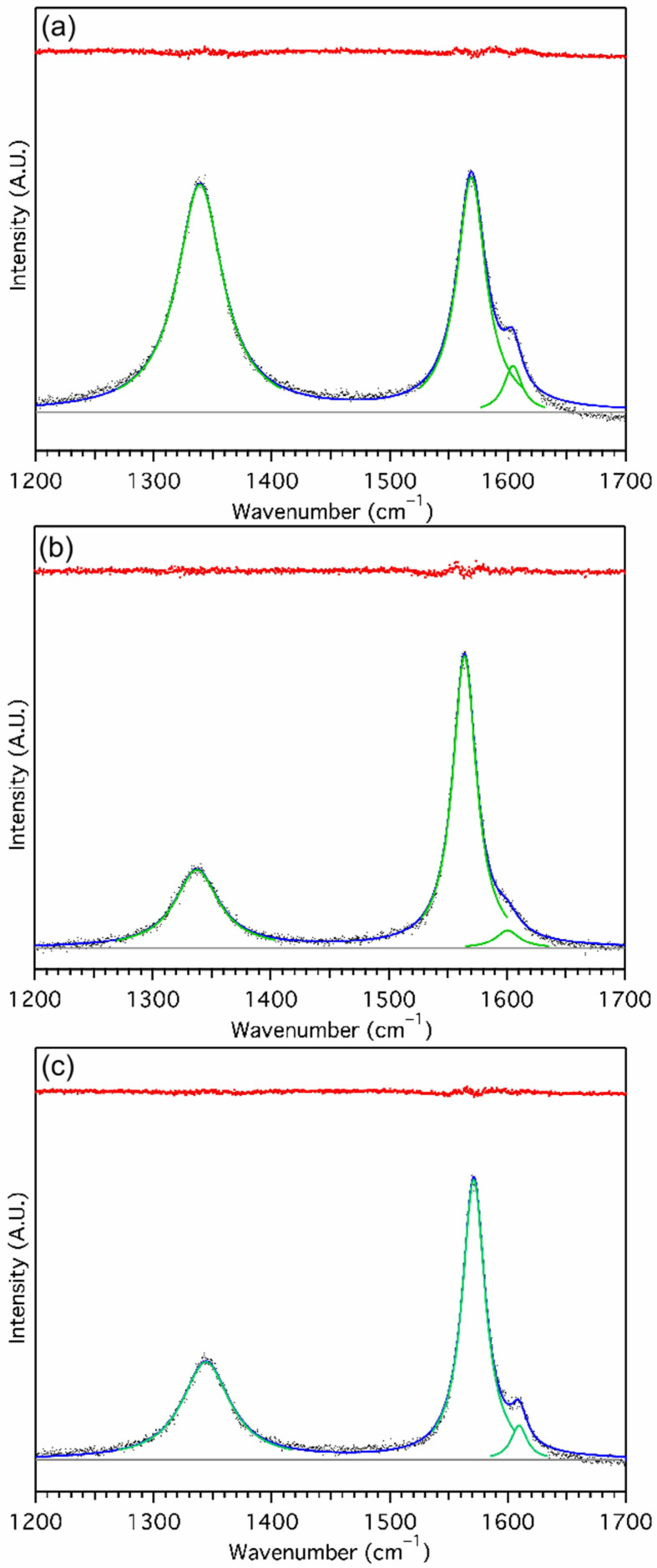
Raman spectra of MGNS from (**a**) NiCl_2_, (**b**) FeCl_2_ and (**c**) CoCl_2_. Data (black dots), peak fits (solid green lines), sum of fits (solid blue line), baseline (solid black line) and residual (red line, above) shown.

Raman spectra show D (1340 cm^−1^) and G (1587 cm^−1^) bands due to sp^3^ and sp^2^ bonded carbon, respectively, and a D’ band as a shoulder on the G band. MGNS-Ni displays the highest degree of sp^3^ bonding of the three materials with an I_D_/I_G_ ratio of 1.53 (Figure 16). The relatively small size of the MGNS-Ni shells gives them a much higher curvature than the other MGNS, only possible in graphene sheets by additional sp^3^ character. The I_D_/I_G_ ratio of MGNS-Fe (0.49) is much lower as expected, with a significant fraction of the sample composed of multilayer graphene sheets, while MGNS-Co has an intermediate I_D_/I_G_ ratio (0.73) due to the large size (lower curvature) of its nested shells.

### 3.6. Electrochemical Testing

**Figure 17:**
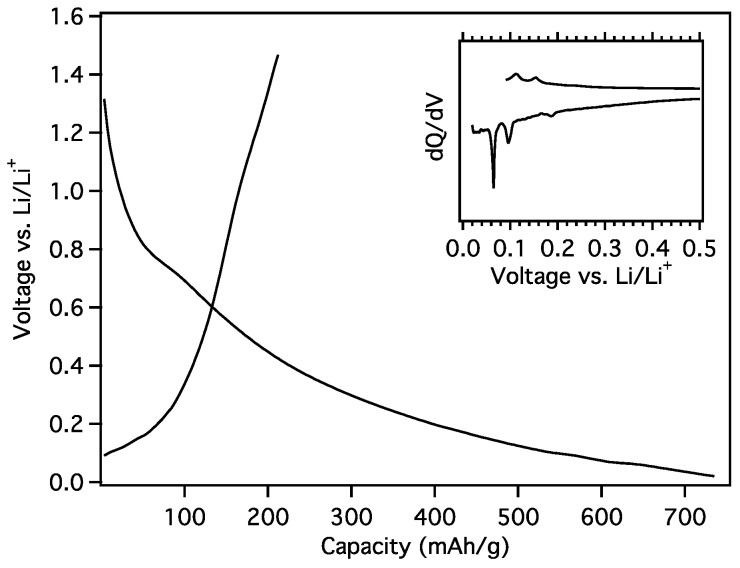
Galvanostatic first cycle MGNS-Ni capacity and differential capacity (inset) plots.

The onset of initial loading (lithiation) of MGNS-Ni occurred near 1.0 V vs. Li/Li^+^ with a gradual decrease in electrochemical potential to the cutoff potential of 20 mV (Figure 17). A differential plot of the galvanostatic curve shows loading peaks at 185, 95 and 65 mV and unloading peaks 110, 155 and ~240 mV, similar to the staging potentials of graphite intercalation (Figure 17 inset), albeit much broader. The reversible capacity was found to be 212 mAh/g with a first cycle Coulombic efficiency (CE) of 28.9%. The high irreversible capacity can be explained the formation of SEI to passivate the large surface area of the MGNS-Ni.

**Figure 18:**
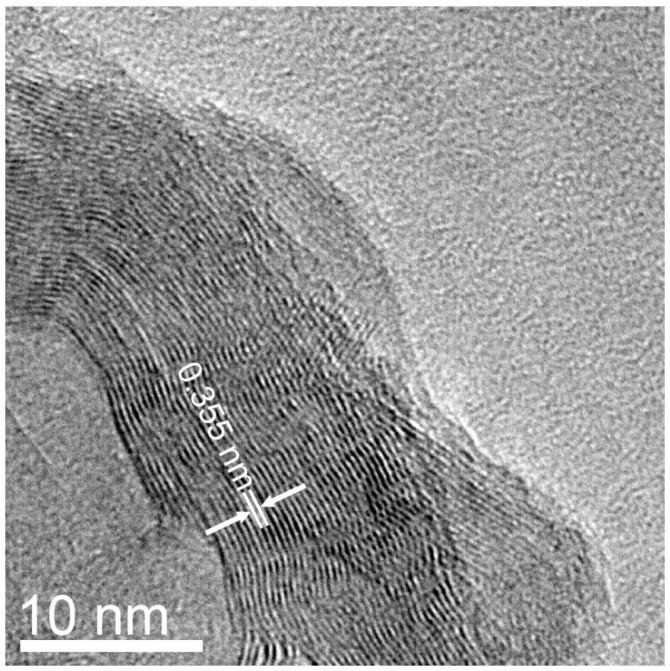
TEM micrograph of MGNS-Ni removed from a cell that was loaded to 0.02 V with (inset) HR-TEM of the lattice. The superimposed arrows and dimension lines show the interwall spacing.

A cell was disassembled and examined by TEM after electrochemically loading an MGNS-Ni with Li. The loaded MGNS-Ni shows an average layer spacing of 3.57 Å, much larger than unloaded MGNS-Ni at 3.4 Å. The layer spacing is consistent with stage-2 (LiC_12_) formation; any stage-1 loading could have been de-intercalated due to the necessity of brief exposure of the electrode to air or the instability in the electron beam of the TEM (Figure 18) [22]. It is clear from the TEM images and the differential capacity curves that MGNS stores Li ion by intercalation between its graphene layers.

**Figure 19:**
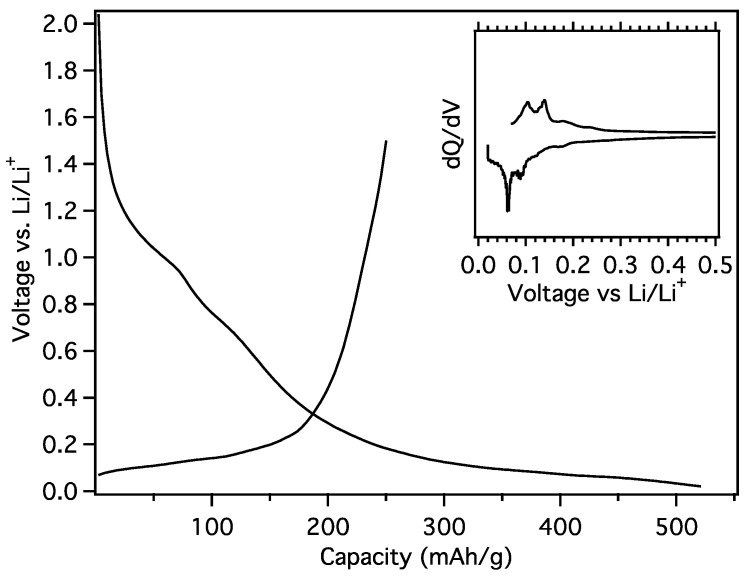
Galvanostatic first cycle MGNS-Fe capacity and differential capacity (inset) plots.

The initial charge transfer to MGNS-Fe occurred at ~1.2 V, which is characteristic of carbonate solvent reduction to form the SEI (Figure 19). The potential steeply decreased to 200 mV, thereafter decreasing with a more moderate slope to the 20 mV cutoff potential. The differential capacity plot of the galvanostatic loading shows peaks at 180, 120, 90 and 60 mV. Two distinct charge transfer peaks at 100 mV and 120 mV and two smaller peaks at 180 mV and 240 mV are evident during unloading. The reversible capacity was found to be 250 mAh/g with a first cycle CE of 46.3%.

**Figure 20:**
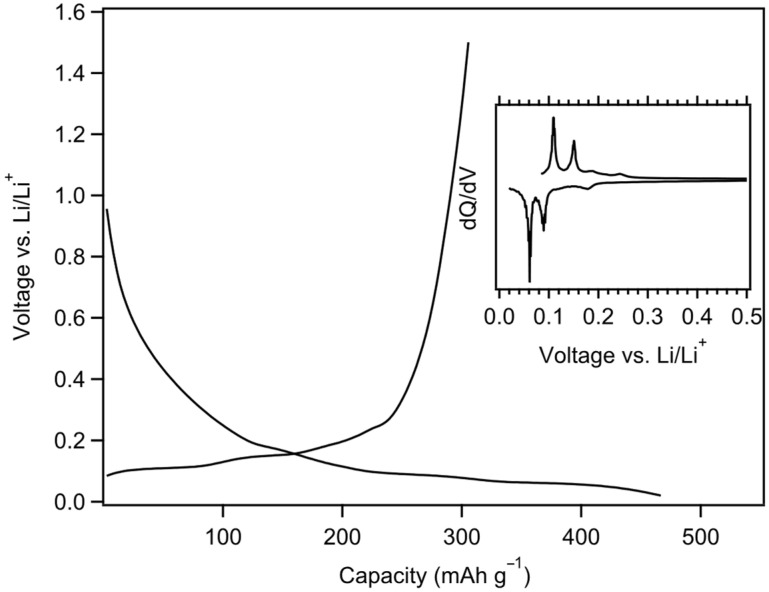
Galvanostatic first cycle MGNS-Co capacity and differential capacity (inset) plots.

The onset of MGNS-Co 1st cycle loading occurs at ~1 V and gradually decreases during the SEI formation process. Li intercalation peaks in the differential capacity plot can be observed at 180, 120, 95 and 60 mV and de-intercalated at 105, 150, 180 and 240 mV (Figure 20). The peaks are much sharper than observed in the other materials, particularly the de-intercalation peaks, more similar to the staging behavior observed in graphite. Reversible capacity was found to be 306 mAh/g with a 1st cycle CE of 66.7%, rising to 320 mAh/g and 100% CE by cycle 10.

The increase in reversible capacity with the change of the metal catalyst from Ni to Fe and Co could in part be due to the change in layer spacing observed, or equivalently, the decrease in turbostratic disorder. Reversible capacity of graphitic materials is strongly influenced by the crystallinity, decreasing as the degree of turbostratic disorder increases. The larger size of the Co-MGNS allows a greater fraction of its walls to run parallel, adopting a more graphitic structure with nested graphene layers. In addition, the expansion of the wall spacings upon loading becomes a smaller fraction of the MGNS size, reducing the associated stress and thus accommodating a higher degree of lithiation.

It is interesting to note that the initial CE of the MGNS materials improves as the surface area declines, but not in a proportionate fashion. Decreasing the surface area from 155 m^2^/g (MGNS-Ni) to 70 m^2^/g (MGNS-Fe) increases the CE from 28.9 to 46.3%, decreasing the irreversible capacity from 521 to 290 mAh/g. A much smaller further fractional decrease to 60 m^2^/g (MGNS-Co) results in a proportionally much larger increase in CE, from 46.3 to 66.7%, and a decrease in irreversible capacity from 290 to 143 mAh/g. One possible explanation is that the interior of the MGNS-Co might be more effectively blocked from electrolyte access. Many of the holes in the shells through which the metal catalyst was removed could become blocked by SEI formation, limiting access to the interior. MGNS-Co, being much larger than the other materials, have a much larger shell area to hole area ratio, and thus a much smaller fraction of their interior space could be accessible to the electrolyte for SEI formation.

**Figure 21:**
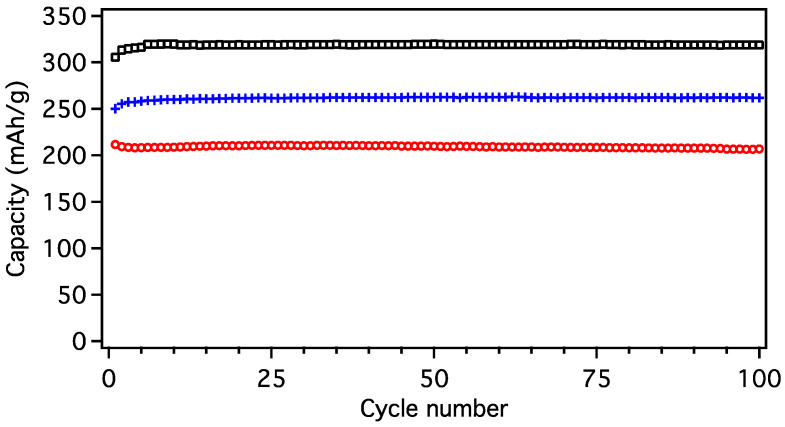
Plot of reversible capacity over 100 load/unload cycles for MGNS-Ni (red/circles), MGNS-Fe (blue/crosses) and MGNS-Co (black/squares).

No capacity fade was observed in any MGNS during 100 cycles. The CE of the cells rose to 100% by cycle 10 and the reversible capacity of MGNS-Fe and MGNS-Co the capacity increased to 260 and 320 mAh/g after 10 cycles, respectively, while MGNS-Ni remained stable at 206 mAh/g for 100 cycles (Figure 21). MGNS-Co was further tested, retaining 100% of 1st cycle capacity (306 mAh/g) through 412 cycles (Figure S2).

### 3.7. Electrochemical Cycling in Propylene Carbonate

**Figure 22:**
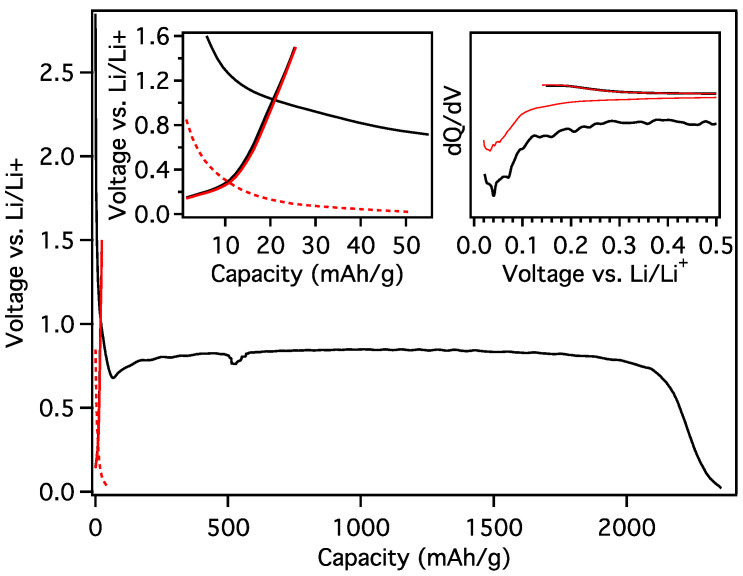
Galvanostatic load and unload of MGNS-Fe cycled in PC for (black) First cycle and (red) second cycle with (right inset) their respective differential capacity plots.

Cells of each MGNS material were assembled and tested in PC with 1 M LiPF_6_ supporting electrolyte. Galvanostatic load curves of MGNS-Fe electrodes displayed a plateau at 800 mV, PC decomposition and active material destruction in graphite (Figure 22) [8]. The reversible capacity was found to be 25 mAh/g, far less than obtained in EC:DMC (260 mAh/g). The low reversible capacity in PC is consistent with TEM observations that the material is primarily composed of multilayer graphene sheets with only a small fraction of closed shells, the former of which would be expected to exfoliate, like graphite, in PC. It is tempting to estimate the fraction of closed shells to be ~10% from the ratio of the reversible capacities observed in PC and EC:DMC; however, the fraction of closed shells may be significantly higher as some may be electrically isolated by the physical disruption of the electrode caused by exfoliation and SEI layer formation.

**Figure 23:**
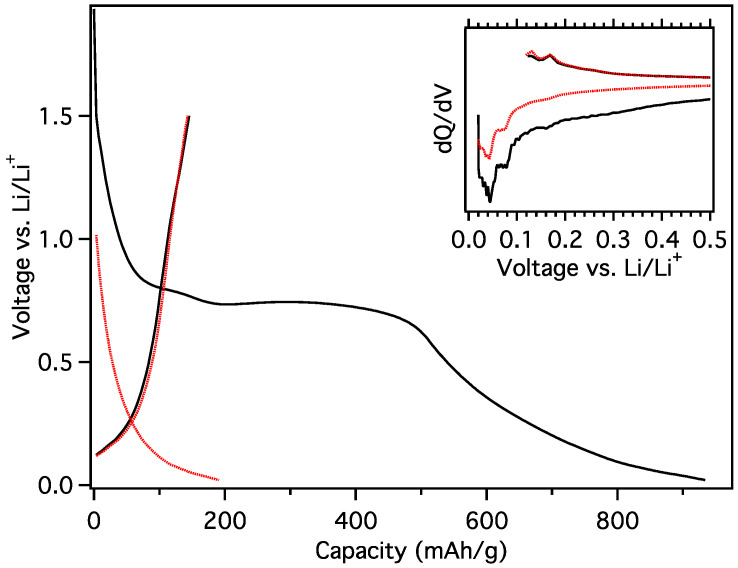
Galvanostatic load and unload of MGNS-Ni cycled in PC for (black) First cycle and (red) second cycle with (inset) their respective differential capacity plots.

A plateau at 800 mV was also observed during the 1st load of MGNS-Ni in PC; however, it was followed by another plateau ~770 mV and eventually reached the cut off potential (Figure 23). Intercalation and de-intercalation peaks can be seen in the differential capacity; however, they are much less well defined than those that occur in EC:DMC. The reversible capacity was found to be 150 mAh/g which is much larger than the MGNS-Fe sample, but significantly lower than 200 mAh/g obtained in the ethylene carbonate-based solvent. The nested graphene shells of MGNS-Ni should be difficult to exfoliate. However, while multilayer graphene sheets such as those of MGNS-Fe were not observed in TEM, some portion of the material may have been incomplete shells that would allow exfoliation, thus decreasing the reversible capacity in PC.

**Figure 24:**
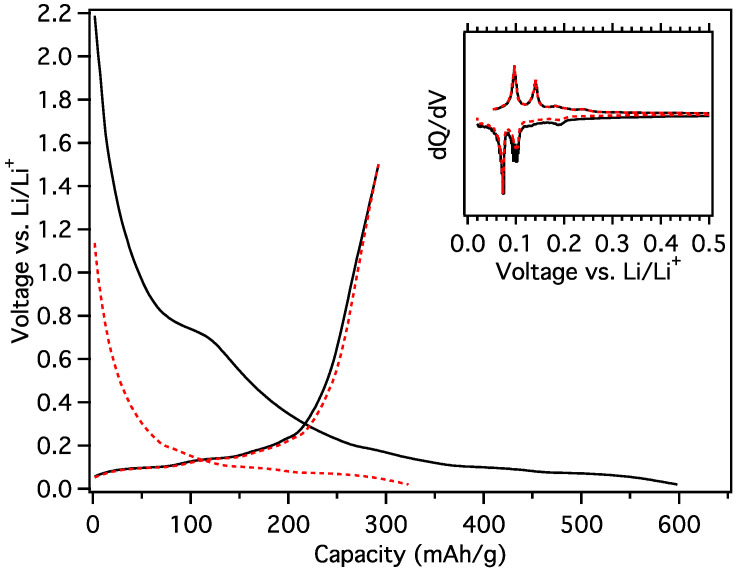
Galvanostatic load and unload of MGNS-Co cycled in PC for (black/solid) first cycle and (red/dash) second cycle with (inset) their respective differential capacity plots.

**Figure 25:**
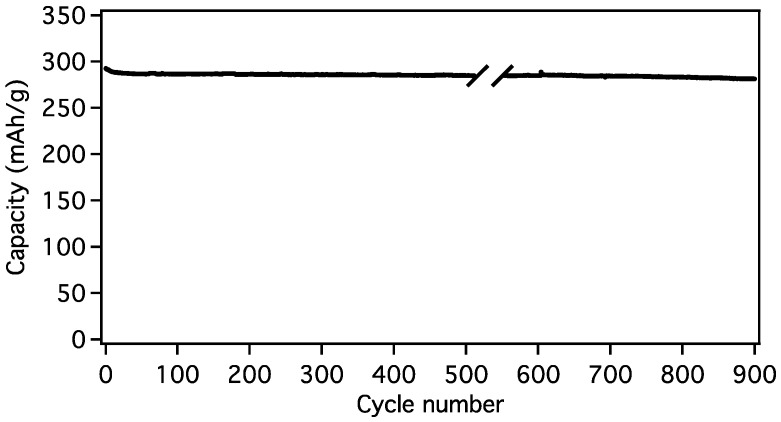
Cycle life of MGNS-Co in 1 M LiPF6 propylene carbonate solvent. The cycles separated by dashed lines and devoid were performed at low temperatures (see below).

In contrast to the other materials, only a small plateau occurs at 770 mV during the 1st load of MGNS-Co. Sharp insertion and removal peaks indicative of lithium staging are readily observed in the galvanostatic differential (Figure 24). The initial reversible capacity was 292 mAh/g, somewhat smaller than obtained in the EC:DMC. A slight decrease in capacity was observed over the first few cycles (288 mAh/g after 20 cycles); however, the cell remained exceptionally stable for 510 cycles, having lost only 2.6% of its initial capacity when it was transitioned to low temperature testing (Figure 25).

**Figure 26:**
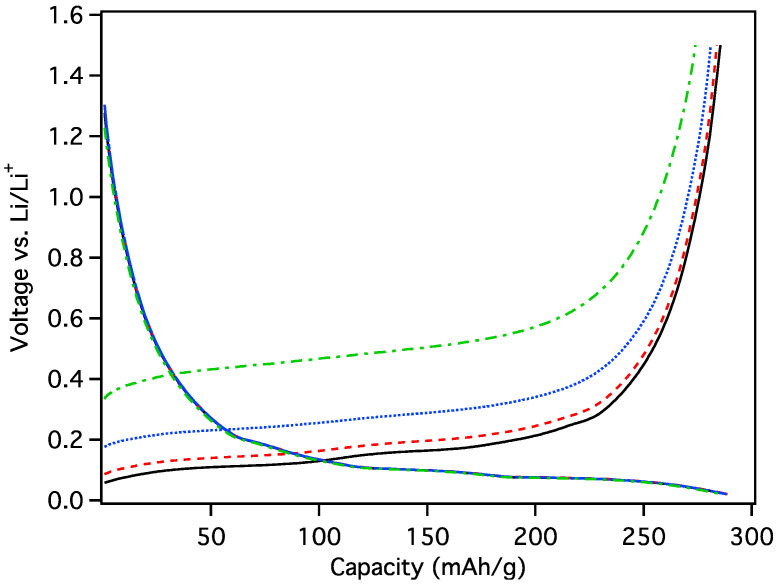
Galvanostatic plots of MGNS-Co unload curves at −5 °C (solid black), −15 °C (red dashes), −25 °C (blue dots), and −35 °C (green dots and dashes) after loading at 25 °C.

**Figure 27:**
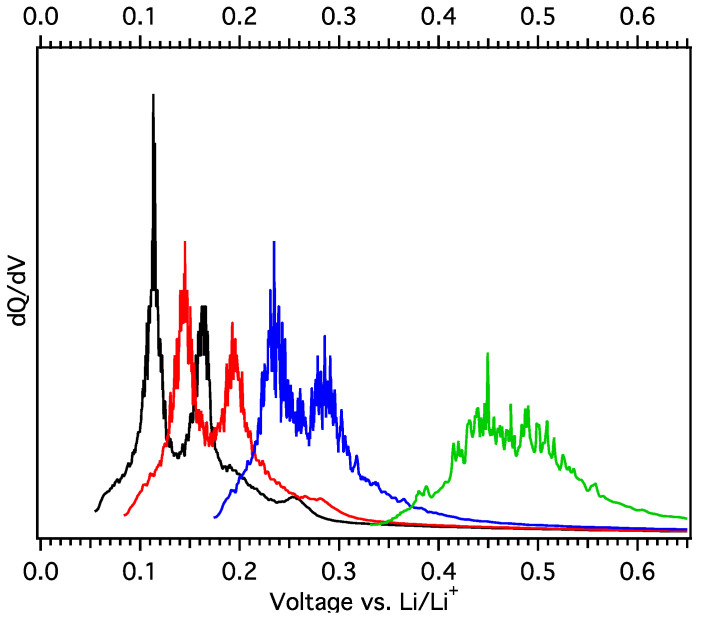
Differential capacity plots of the MGNS-Co unload curves shown in Figure 26 at −5, −15, −25, and −35 °C (from left to right, black, red, blue and green, respectively).

**Figure 28:**
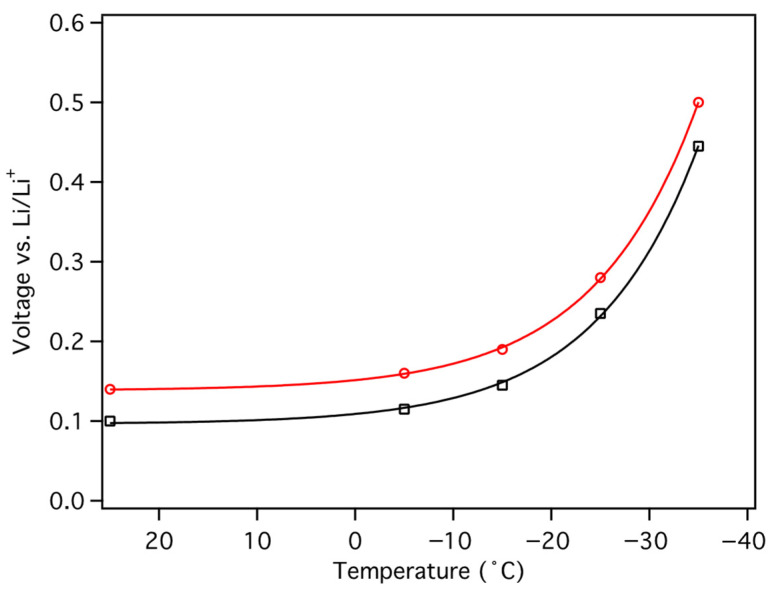
Stage 1 (black squares) and Stage 2 (red circles) de-intercalation unload potentials as function of temperatures. Solid lines are fit to an exponential function with coefficients *A*, *B*, and *C* of 0.096(3), 0.013(1) and 0.095(4) for Stage 1 and 0.138(2), 0.0131(9) and 0.95(2) for Stage 2, where the parenthetical numbers are the standard deviations in the final digits.

Constant current (C/5) cycling at low temperatures was performed by 25 °C loading and subsequent unloading at −5 °C, −15 °C, −25 °C, and −35 °C, each for a total of 4 cycles (Figure 26). The RT galvanostatic loading plots are essentially identical, as one might expect since they were all performed at RT, and cell cycling at −5 °C was very similar to that at RT, but with a modest 15 to 20 mV increase in unload potential (Figure 24 and Figure 26). The unload potential progressively increased as unload temperature was decreased (Figure 26 and Figure 27). It should be noted that the load potential increased exponentially, fitting well to

(2) $$Potential=A+Be^{-Cx}$$

as would be expected given the functional form of the solvent viscosity temperature dependence (Figure 28) [23]. However, the cell was able to obtain 95% of the RT reversible capacity at −35 °C, limiting the overall decrease in energy density to less than ~15%.

**Figure 29:**
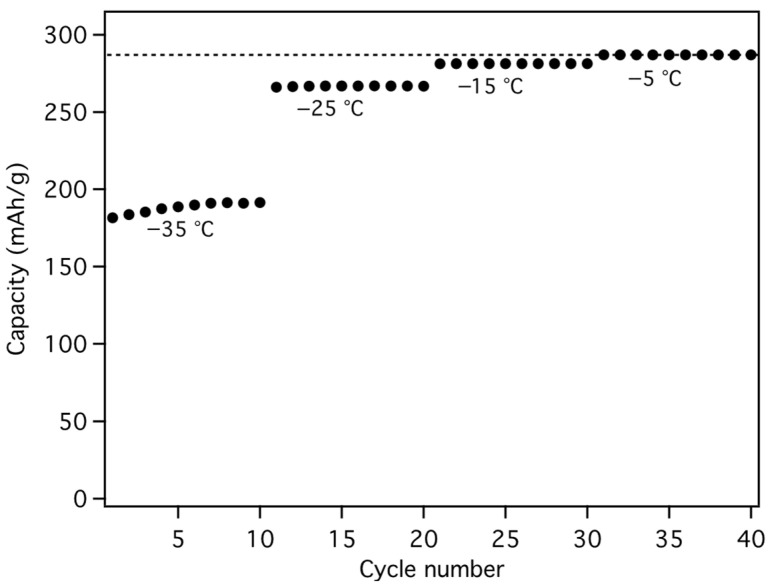
Capacity of MGNS-Co when loaded and unloaded with a CC/CV and CC (C/5) regime, respectively, at the indicated temperatures. The RT reversible capacity is indicated by the dotted line.

A constant current followed by constant voltage (CC/CV) cycling regime was used to test Li-insertion capabilities at low temperature. This eliminates premature load cutoff caused by overpotential and better exemplifies real world Li-ion battery charging. A CC load (C/5) was performed with a 0.02 V cutoff and then a CV (0.02 V) was applied until 12% of the original current was obtained. The subsequent CC (C/5) unload was performed at the same temperature as loading. The total insertion times, in the absence on external heating, at −35, −25, −15, and −5 °C were 12.8, 9.3, 6.4, and 5.7 h, respectively, which are acceptable for “overnight” charging times in most northern regions. The percent of the total loading time spent at CC for each temperature was 5.9, 18.8, 62.5, and 85.5%, respectively. At −5 °C, the cell obtained the same reversible capacity as at RT, while at −15, −25, and −35 °C it obtained 98, 93, and 66% of the RT capacity (Figure 29).

After testing at low temperatures, the cell was returned to the cycling regime at RT. No decrease in capacity was observed after the LT tests and the cell continued to cycle stably with minimal fade, losing just 3.8% of its total capacity over 900 cycles (Figure 25).

### 3.8. High-Rate Loading/Unloading

**Figure 30:**
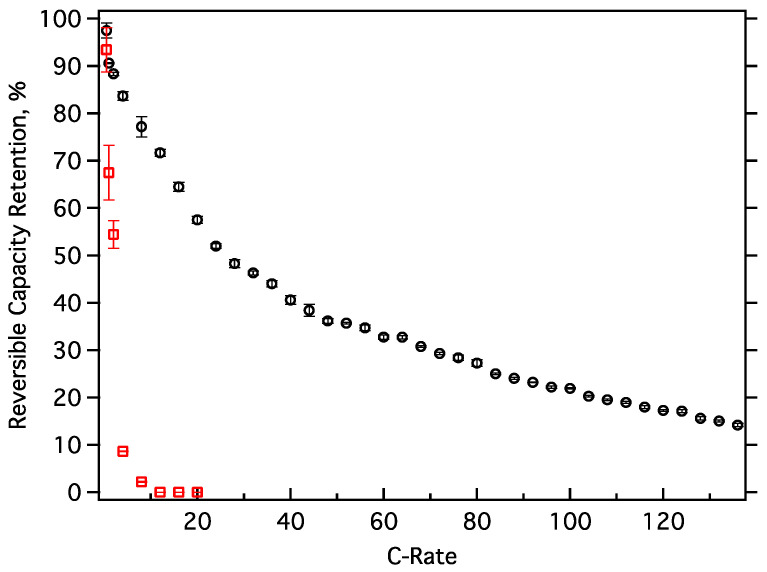
High-rate testing of MGNS-Ni (black circles) and SFG-15 graphite (red squares). Capacities at each rate are an average of 3 cycles with respective standard deviations shown by error bars.

Commercial graphite is incapable of rapid loading due to long solid-state diffusion distances, typically 5–15 microns, required for complete Li-ion intercalation. The total intercalation distance of MGNS-Ni is less than 100 nm, the outer circumference of the shells, three orders of magnitude shorter than graphite, potentially allowing much more rapid Li-ion loading and unloading. Cells of SFG-15 (commercial synthetic graphite) and MGNS-Ni were prepared and cycled using a constant current regime, with identical load and unload rates, 3 times at each progressively higher rate. SFG-15 cells were unable to intercalate meaningful capacities of lithium beyond a 2 C rate (Figure 30). In comparison, MGNS-Ni is able to load more than 55% of its full (C/4) capacity at 20 C rate; even at rates as high as 136 C it was still able to load 15% of its capacity. At a 100 C rate, MGNS-Ni can load and unload 47 mAh/g in <8 s, a power density of ~60 kW/kg (3.7 V cell, materials basis), similar to electrochemical capacitors but with much higher energy density. High-rate cycling had no discernable deleterious effect on the MGNS-Ni. Full C/2 rate capacity was obtained after completion of the high-rate testing.

### 3.9. Variation of Metal Salt Percentage

Changing the catalysts from Ni to Fe to Co chlorides, while maintaining a mass fraction of 0.2, has a dramatic effect on the properties of the MGNS produced as described above. Variation of the mass fraction of catalyst used in the range of 0.025 to 0.40 also significantly, albeit somewhat less dramatically, affects the resultant properties.

**Figure 31:**
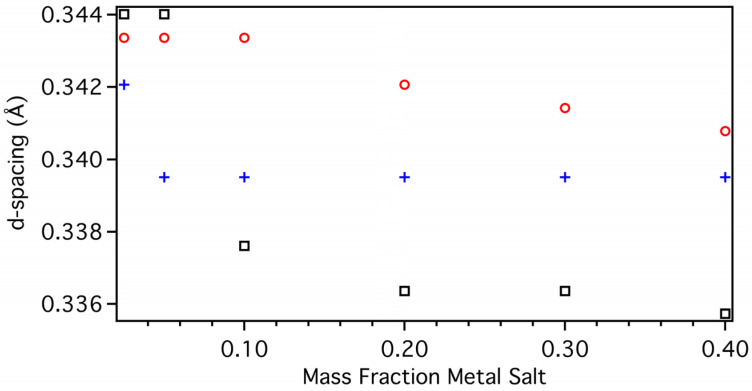
The d-spacing of the 002 reflections of MGNS-Ni (red/circles), MGNS-Fe (blue/crosses) and MGNS-Co (black/squares) as a function of metal salt mass fraction.

At the lowest mass fraction examined, the interwall spacing exceeds 3.4, regardless of catalyst (Figure 31). The materials produced at this low mass fraction were previously shown to be carbon nanochains (CNCs) rather than MGNS [24,25]. Increasing the mass fraction used for MGNS-Fe to 0.05 results in the production of graphitic material in addition to CNCs, presented as a broad reflection as previously reported (Figure S3) [25]. MGNS-Fe made with mass fractions of 0.1 and larger present a single reflection of 52% 3D graphitic order (Figure S4) and near constant width indicating little or no wall growth. When synthesized with Ni catalyst, the product is CNCs up to mass fractions of 0.1, the d-spacing remaining 0.343 and 3D graphitic order of 7%, with MGNS formation indicated by an abrupt increase in d-spacing to 0.342 (23% 3D graphitic order) at a mass fraction of 0.2 (Figure 31, Figure S4 and S5). Further increases to the mass fraction to 0.3 results in the appearance of a second reflection as a shoulder at a d-spacing of 0.336 (89% 3D graphitic order) indicating the production of a more graphitic material in addition to MGNS-Ni, which becomes the majority phase at a mass fraction of 0.4. The width of the MGNS-Ni reflection is invariant, indicating that the wall thickness is constant and independent of catalyst mass fraction. Using a Co catalyst, CNCs result in mass fractions of 0.05 and less with a d-spacing of 0.344, transitioning to a primarily MGNS phase at mass fraction of 0.1 (Figure 31, Figure S4 and S6). The reflection width and d-spacing of the MGNS-Co decrease further with increasing mass fraction, indicating increasing wall thickness and 3D graphitic order, which increases to 96% at mass fraction of 0.4.

## 4. Conclusions

In summary, MGNS were prepared from cellulose, a sustainable biopolymer, and either Ni or Co salt catalyst at mass fractions of 0.2 or greater, while Fe salt primary gave multilayer graphene. At lower mass fractions, CNC or CNC/MGNS mixtures were formed with all catalysts. The MGNS dimensions obtained can be selected by changing the metal catalyst salt and/or its mass fraction in the synthesis. MGNS are roughly spheroidal, most of which are distorted, with relatively large fractions of the walls being flat rather than curved. MGNS-Ni appear to be ~30–50 nm in diameter, roughly spheroidal and exhibit minimal size and wall thickness change with catalyst mass fraction. Far larger MGNS were formed with Co, some with diameters of 1 µm or more, as seemingly flexible, hollow shells.

MGNS reversibly cycles Li-ions by an intercalation mechanism similar to graphite. The reversible gravimetric capacity of each MGNS prepared from different metal salts correlates well to its degree of 3-D graphitic order, with MGNS-Co obtaining the highest, 320 mAh/g, ~90% of that typical of commercial graphite in EC:DMC electrolyte. While MGNS-Ni, the smallest of the MGNS, exhibited the lowest reversible gravimetric capacity (212 mAh/g), it proved capable of very high charge/discharge rates (136 C or greater) with little to no capacity fade, presumably due to the small diffusion distances. The nested-shell structure prevents exfoliation that can occur when cycling in propylene carbonate. This enables cycling in a PC-only solvent-based electrolyte, with stable cycling with only 3.8% capacity fade over 900 cycles and high capacities at temperatures as low as −35 °C. At this very low temperature, 95% of the RT reversible capacity is retained for MGNS-Co, with only a modest charge potential increase due to the increase in viscosity of the solvent. In summary, MGNS is a carbon negative material produced from inexpensive biomass that obtains extremely fast charge rates, long-cycle life and excellent low temperature performance in low-cost electrolytes, mitigating the environmental impact of the graphite it would replace while potentially reducing the cost of Li-ion cells.

## Data Availability

The original contributions presented in this study are included in the article/Supplementary Material. Further inquiries can be directed to the corresponding authors.

## Supplementary Materials

The following supporting information can be downloaded at: https://www.mdpi.com/article/10.3390/ma18163918/s1, Figure S1: GGA thermograms of Ni-MGNS and Co-MGNS conducted in air at a rate of 20 °C/min; Figure S2: Plot of reversible capacity over 400 load/unload cycles for MGNS-Co and Coulombic efficiency; Figure S3: The (002) reflection of MGNS-Fe as a function of catalyst mass fraction; Figure S4: Fractional graphitic order of MGNS-Ni, MGNS-Fe and MGNS-Co as a function of catalyst mass fraction; Figure S5: The (002) reflection of MGNS-Ni as a function of catalyst mass fraction; Figure S6: The (002) reflection of MGNS-Co as a function of catalyst mass fraction.
