# Supplementary material for: Multilayer Graphene Nanoshells from Biomass for Fast-Charge, Long-Cycle-Life and Low-Temperature Li-Ion Anodes
Source: Materials (Basel). 2025 Aug 21;18(16):3918. doi: 10.3390/ma18163918 (PMC12387679; doi:10.3390/ma18163918)
Supplement: Supplementary file 1 [file materials-18-03918-s001.zip › materials-3823434-supplementary.pdf]

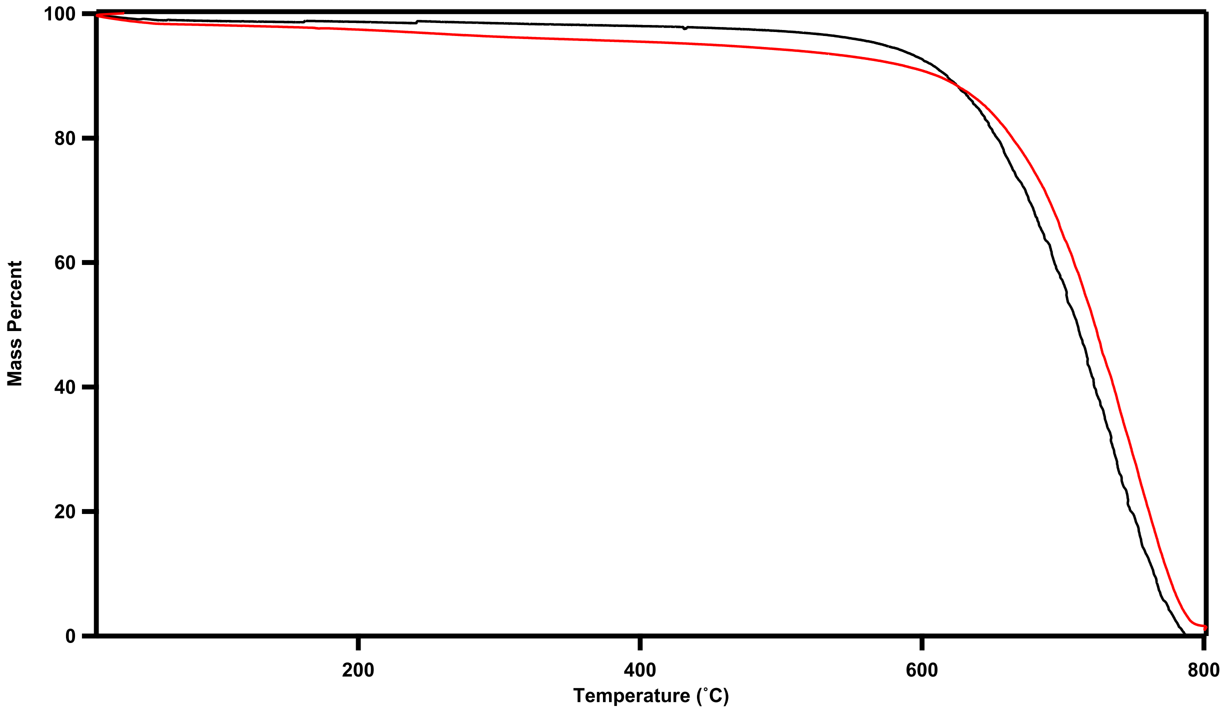

Figure S1 - TGA thermograms of Ni-MGNS (red) and Co-MGNS (black) conducted in air at a rate of 20 ˚C/min.

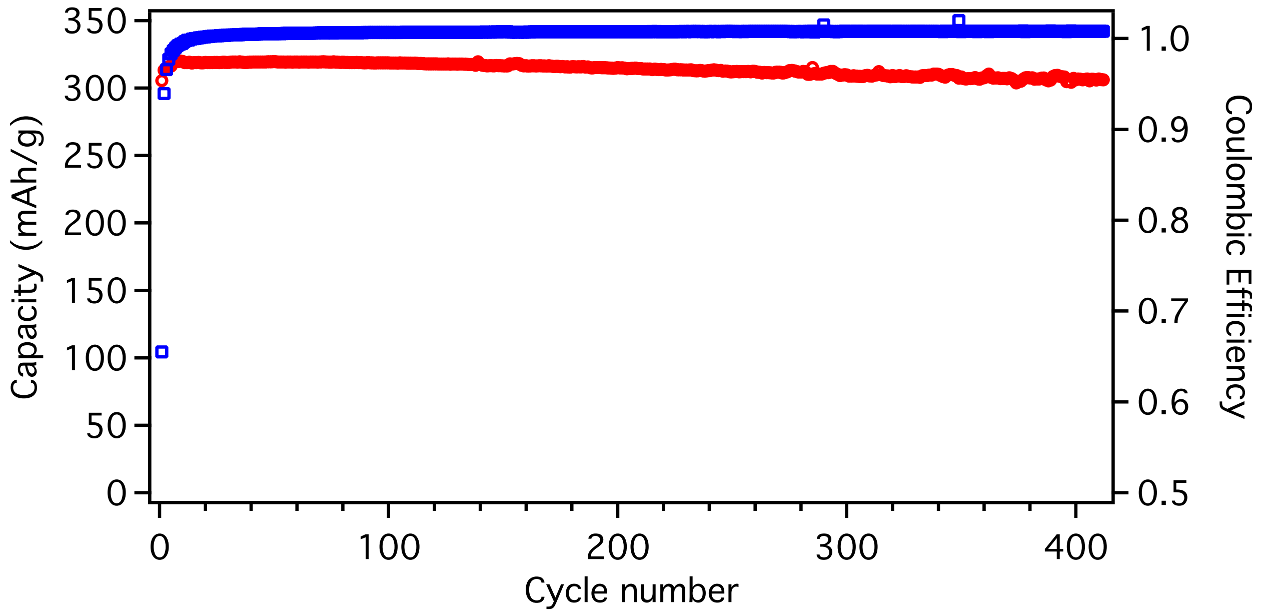

Figure S2 - Plot of reversible capacity over 400 load/unload cycles for MGNS-Co (red/circles, left axis) and Coulombic efficiency (blue/squares, right axis).

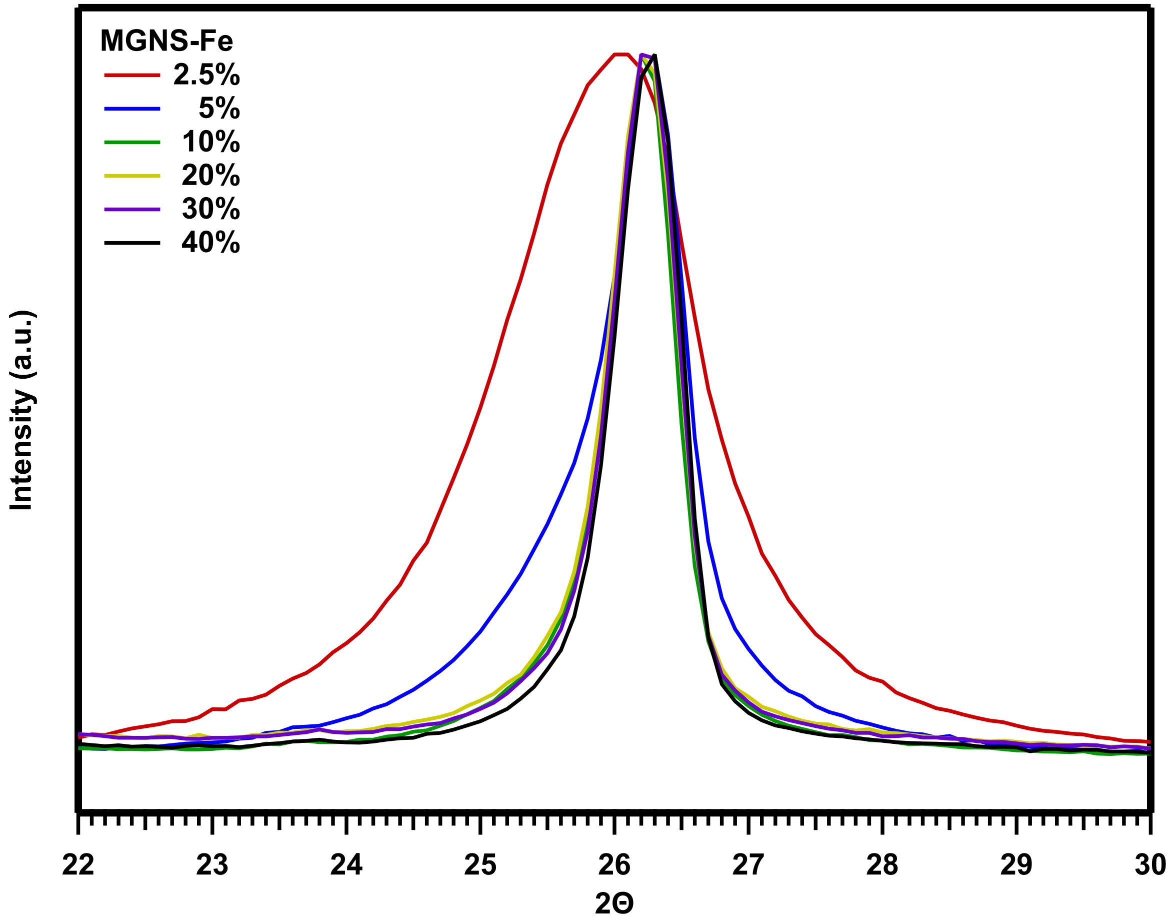

Figure S3 - The (002) XRD reflection of MGNS-Fe as a function of catalyst mass fraction (see legend in figure).

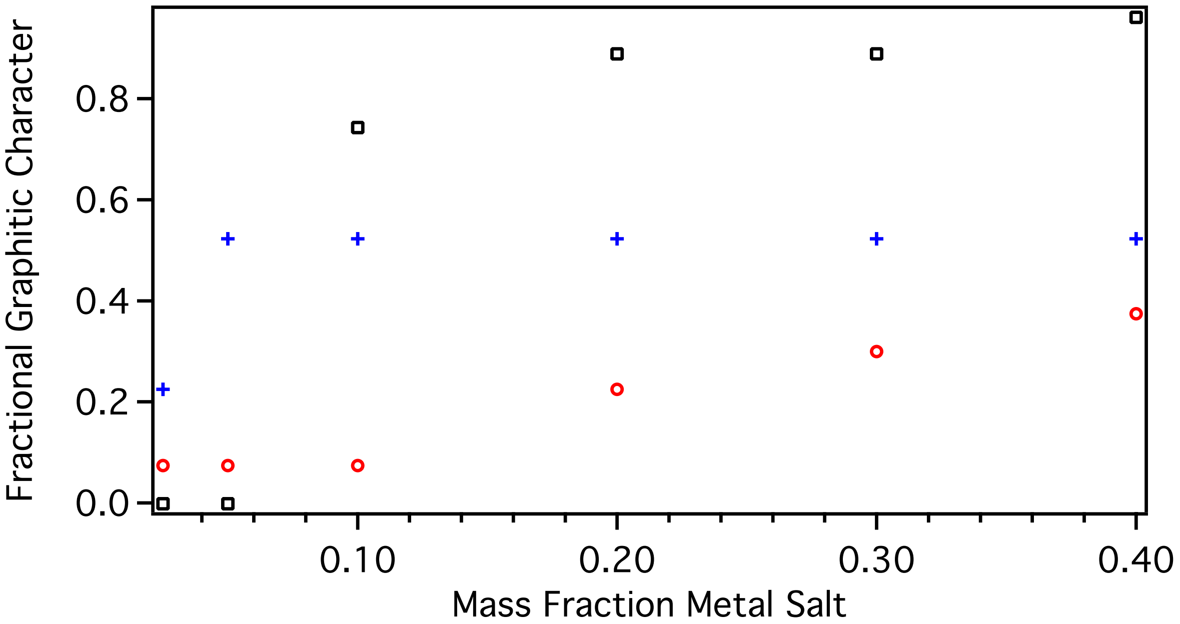

Figure S4 - Fractional graphitic order of MGNS-Ni (red/circles), MGNS-Fe (blue/crosses) and MGNS-Co (black/squares) as a function of catalyst mass fraction.

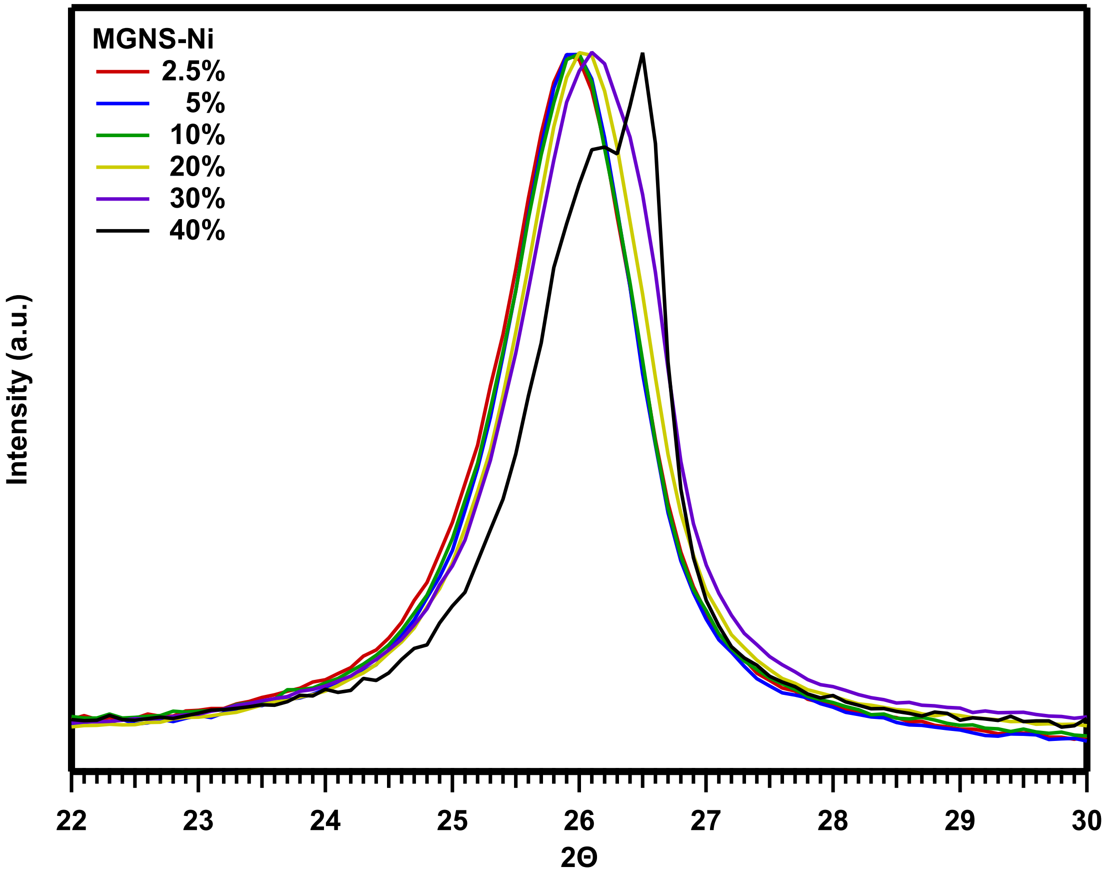

Figure S5 - The (002) XRD reflection of MGNS-Ni as a function of catalyst mass fraction (see legend in figure).

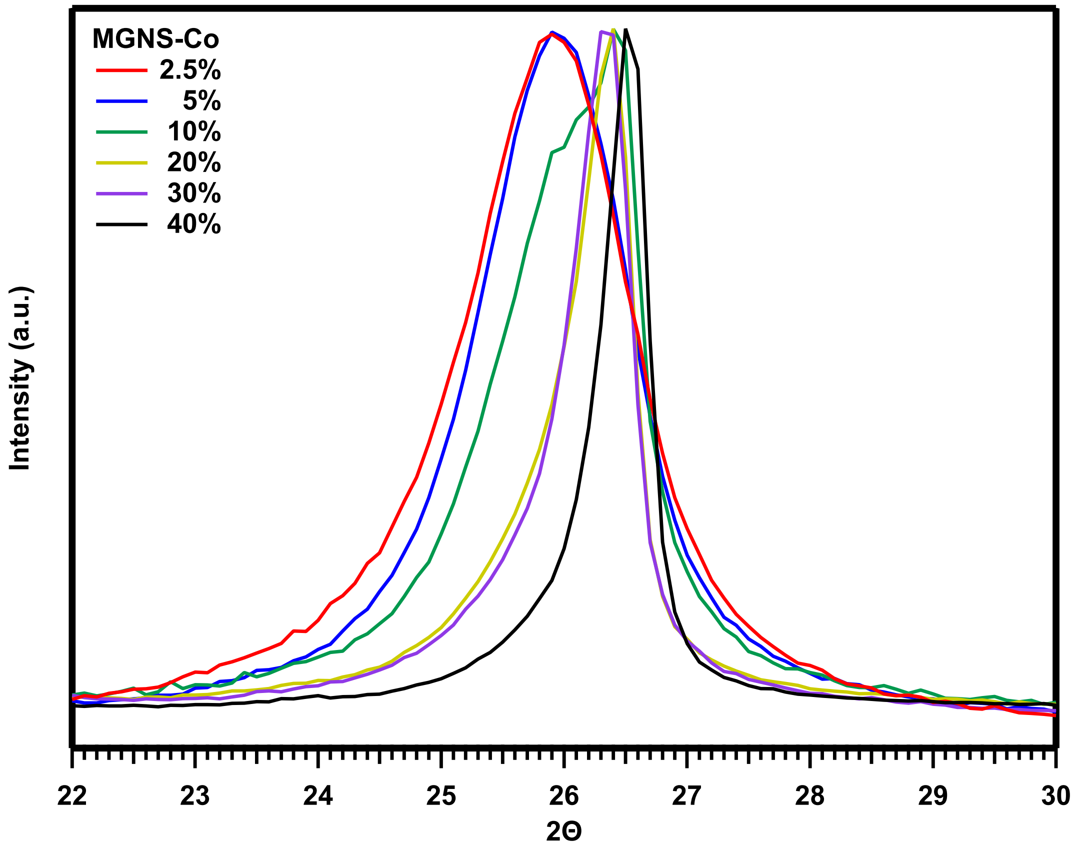

Figure S6 - The (002) XRD reflection of MGNS-Co as a function of catalyst mass fraction (see legend in figure).
